# Stroke at baseline of the Brazilian Longitudinal Study of Adult Health (ELSA-Brasil): a cross-sectional analysis

**DOI:** 10.1590/1516-3180.2018.0129060818

**Published:** 2018-10-22

**Authors:** Fernanda Gabriela de Abreu, Alessandra Carvalho Goulart, Marina Gabriela Birck, Isabela Martins Benseñor

**Affiliations:** I Master’s Student, Faculdade de Medicina da Universidade de São Paulo (FMUSP), São Paulo (SP), Brazil.; II MD, PhD. Assistant Professor, Centro de Pesquisa Clínica e Epidemiológica, Hospital Universitário, Universidade de São Paulo (USP), São Paulo (SP), Brazil.; III DPH. Master’s Student, Faculdade de Medicina da Universidade de São Paulo (FMUSP), and Associate Professor, Department of Internal Medicine, FMUSP, São Paulo (SP), Brazil.; IV MD, PhD. Associate Professor, Department of Internal Medicine, School of Medicine, Universidade de São Paulo (USP), and Assistant Professor, Centro de Pesquisa Clínica e Epidemiológica, Hospital Universitário, Universidade de São Paulo, São Paulo (SP), Brazil.

**Keywords:** Cerebrovascular disorders, Stroke, Risk factor, Secondary prevention

## Abstract

**BACKGROUND:**

Secondary prevention of stroke is a very important goal for achieving continuous reduction in stroke mortality rates over the next decades.

**DESIGN AND SETTING:**

Cross-sectional analysis on the Brazilian Longitudinal Study of Adult Health ­(ELSA-Brasil), with data from Salvador, Vitória, Belo Horizonte, Rio de Janeiro, São Paulo and Porto Alegre.

**METHODS:**

This descriptive analysis focused on secondary prevention of stroke among participants who self-reported a medical diagnosis of stroke at the baseline of ELSA-Brasil, and its association with sociodemographic characteristics.

**RESULTS:**

Overall, 197 participants (1.3%) reported a prior medical history of stroke. Participants with stroke were older and less educated and had lower mean monthly family income, compared with non-stroke participants. Among all stroke cases, 23.7% did not use any medication for secondary prevention of stroke. Use of secondary prevention was higher among men than among women (respectively, 59.6% versus 40.4%; P = 0.02 for aspirin; and 71.4% versus 28.6%; P = 0.04 for other antiplatelet drugs). Having private health insurance was associated with greater use of less cost-effective and more expensive medications (like angiotensinogen receptor blockers) and a tendency to use antiplatelet drugs other than aspirin, among participants reporting stroke, compared with others. Use of medication decreased as time passed after suffering a stroke.

**CONCLUSIONS:**

In this sample of individuals with better access to healthcare services, use of secondary prevention for stroke was low, which may suggest that the situation in the general population is worse. Sex was the most important sociodemographic variable associated with low use of secondary prevention.

## INTRODUCTION

Despite the declining stroke rates over recent years, with a reduction of 21% from 2005 to 2015 (19.2% to 22.8%),^1^ stroke remains one of the leading causes of mortality worldwide, particularly in developing countries.^1^^,^^2^ In Brazil, early mortality (at ages of less than 70 years) has presented an impressive decline since 2005, from 55.7% in that year to 30.5% in 2015, but is still very high.^3^ Although the risk of death due to stroke is decreasing in all regions of Brazil, faster declines were observed in the wealthiest areas, thus exacerbating the social inequalities in the country.^4^^,^^5^

Secondary prevention of stroke mortality is a key point in relation to achieving continuous reduction in stroke mortality over the next decades. However, few studies in Brazil provide information about use of secondary prevention. In Joinville, a city in southern Brazil, out of 594 patients who were discharged from public hospitals after their first stroke, 54% did not return to a public or private hospital unit for follow-up at least twice a year; 5.7% (34/594) stated that they were not taking any secondary preventive treatment, had no intention of coming back for secondary prevention, or had no intention of taking the hospital prescription; 8.4% (50/594) looked for private care or for public healthcare units outside their health district; and 8.4% (50/594) were lost, despite thorough searching for them.^6^ In another study in Pelotas (also in the southern region), the rate of use of aspirin for secondary prevention of stroke, angina pectoris and myocardial infarction was 34.3%, i.e. well below the recommended levels for prevention of cardiovascular diseases.^7^

Considering the paucity of studies evaluating secondary prevention for stroke in Brazil, we sought to conduct a cross-sectional analysis on the frequency of secondary prevention of stroke and associated sociodemographic risk factors, using data from participants who reported having a previous medical diagnosis of stroke at the baseline examination of the Brazilian Longitudinal Study of Adult Health (ELSA-Brasil).

In this context, we performed a cross-sectional analysis with the main objective of evaluating secondary prevention of stroke among the participants of the Brazilian Longitudinal Study of Adult Health (ELSA-Brasil), using self-reported information on previous stroke that was obtained at the baseline examination.

## METHODS

### Study design and participants

ELSA-Brasil is a cohort study on 15,105 civil servants aged 35 to 74 years living in six cities (Belo Horizonte, Porto Alegre, Rio de Janeiro, Salvador, São Paulo and Vitória). It was designed to investigate cardiovascular diseases and diabetes and their risk factors.^8^ The baseline assessment took place between August 2008 and December 2010. Previous reports presented more details about the study design, concept and sampling procedures.^8^^,^^9^^-^^10^

In this analysis, we included all participants for whom self-reported information about stroke (N = 15,102) was available from the baseline assessment. All participants answered a question asking about a previous medical diagnosis of stroke.

Each participant was interviewed at the workplace and made a visit to the research center for clinical examinations, in accordance with standard protocols. The interviews and examinations at each site were conducted by trained personal under strict quality control.^10^

### Variables

For the present analysis, the following sociodemographic variables were considered: age (years), sex, self-reported race (white, mixed race, black, Asian or indigenous), educational attainment (less than high school; high school and some tertiary education; or completed college/university or more), mean monthly family income (≤ US$ 1,245; US$ 1,246 to US$ 3,319; or ≥ US$ 3,320) and having private health insurance (%). Local currency (Brazilian reais, BRL) was converted to United States dollars (USD) at a rate of BRL 2.00 = USD 1.00 in December 2008.

Smoking and alcohol use were categorized as never, past or current.^10^ Leisure-time physical activity was measured by means of the International Physical Activity Questionnaire (IPAQ), in its long form, and the subjects were classified in accordance with the World Health Organization criteria, as physically active, partly active or sedentary. Adherence to medication was assessed using simple questions asking about the frequency of use of medicines.

Anthropometric measurements were obtained using standard protocols. Body mass index was calculated as weight divided by height squared (kg/m^2^). Blood pressure (BP) was taken using a validated oscillometric device, the Omron HEM 705CPINT. Three measurements were taken at one-minute intervals. The mean of the latest two BP measurements was taken to be the value for defining situations of high BP.

Presence of hypertension was defined as situations in which medications to treat hypertension were being used, or of systolic blood pressure ≥ 140 mmHg or diastolic blood pressure ≥ 90 mmHg. Presence of diabetes was defined as situations in which there was a previous medical history of diabetes, medications to treat diabetes were being used, or situations of fasting plasma glucose ≥ 126 mg/dl, two-hour plasma glucose ≥ 200 mg/dl, or HbA1C ≥ 6.5%. Presence of dyslipidemia was defined as situations in which lipid-lowering treatment was being used or of LDL-cholesterol (LDL-c) ≥ 130 mg/dl.^10^ The glomerular filtration rate (GFR) was calculated by means of the Chronic Kidney Disease Epidemiology Collaboration (CKD-Epi) equation.

### Use of medication

All participants were asked about their continuous use of prescription or non-prescription medicines and over-the-counter drugs, including pills or liquid medications. Participants were instructed to bring all their medications to the ELSA-Brasil research centers. Then, all medications taken during the last two weeks were reviewed during the baseline assessment.

The medications used were classified as antithrombotics, including anticoagulants (antivitamin K), heparin and antiplatelet medications; as different classes of antihypertensives, such as diuretics, β-blockers, angiotensin-converting enzyme inhibitors (ACEI), angiotensinogen receptor blockers (ARB), calcium channel blockers (CCB), vasodilators, central action and alpha-blockers; or as lipid-lowering drugs (statins and others). Medication adherence was assessed using these four questions and by asking participants to bring medications for checking in the ELSA centers (as stated above).

Conventional 12-lead electrocardiograms (ECGs) were performed using a digital device (Atria 6100, Cardiac Science Corporation, Deerfield, Wisconsin, USA) with automated readings. The technique used for carotid intima-media thickness (CIMT) measurement in ELSA-Brasil was published previously.^11^ The same protocol was applied at all the sites, using a Toshiba (Aplio XG) with a 7.5 MHz linear transducer. We used MIA software to standardize the reading and interpretation of carotid scans.^12^

### Statistical analysis

Cross-sectional associations with self-reported stroke, the main sociodemographic characteristics, the cerebrovascular risk factors and the use of medications for stroke secondary prevention were analyzed. Categorical variables were presented as absolute counts and proportions and were compared using the chi-square test. Continuous variables were presented as means (± standard deviation, SD) and were compared using one-way analysis of variance (ANOVA); or as medians (with interquartile range, IQR) and were compared using the Mann-Whitney test, as necessary.

The relationships between stroke, main cerebrovascular risk factors and the use of medications for secondary prevention of stroke were tested using logistic regression models. Odds ratios (OR) with their respective 95% confidence interval (95% CI) were presented as crude ratios, as age and sex-adjusted ratios and as ratios with multivariate adjustment including age, sex, education, mean family monthly income and private health insurance.

All the analyses were performed using the Statistical Package for the Social Sciences, version 22 (SPSS Inc., Chicago, Illinois, USA). P-values < 0.05 were considered statistically significant.

### Ethics statement

The ELSA-Brasil study was approved at all six centers by their institutional review boards addressing research on human participants, in accordance with the Declaration of Helsinki (approval number CEP-HU/USP: 1555/16). Written informed consent was obtained from all participants.^8^

## RESULTS

Overall, 197 participants (1.3%) reported having a prior medical history of stroke. The participants with stroke were older and less educated and had lower mean monthly family income, larger waist circumference, higher systolic and diastolic blood pressure measurements and higher frequencies of hypertension, diabetes, dyslipidemia and chronic kidney disease, compared with non-stroke subjects. They reported less frequent alcohol intake than did the participants who did not report having had stroke at the baseline.

The mean CIMT values were higher among subjects who reported having had a stroke than among the other participants without this condition (Table 1). Except for α-blockers, which are not used for stroke participants, all classes of antihypertensive drugs were used at higher frequencies among the stroke participants than among the non-stroke participants (Table 1). Adherence to use of any medication was higher among the stroke participants than among the other participants: 46% among stroke participants versus 37.1% among non-stroke participants (P = 0.02). However, neither of these frequencies of use was very high.

**Table 1.** Sociodemographic and clinical characteristics and use of medication for secondary prevention, according to self-reported occurrences of stroke at the baseline examination of ELSA-Brasil

	Stroke		
	No	Yes	P-value
	n = 14,905 (%)	n = 197 (%)	
Age (years)*	52.0 ± 9.1	58.4 ± 8.9	< 0.0001
Women (%)	8,110 (54.4)	108 (54.8)	0.91
Race (%)			
White	7,700 (52.3)	88 (45.6)	0.41
Mixed	4,143 (28.1)	59 (30.6)	
Black	2,360 (16.0)	37 (19.2)	
Asian	368 (2.5)	6 (3.1)	
Indigenous	154 (1.0)	3 (1.6)	
Education (%)			
Less than high school	1,874 (12.6)	48 (24.4)	< 0.0001
High school and some college/university	5,162 (34.6)	69 (35.0)	
College/university or more	7,869 (52.8)	80 (40.6)	
Mean family monthly income in US$ (%)			
≤ 2,489.00	3,923 (26.4)	71 (36.4)	0.006
2,490.00-6,639.00	5,648 (38.1)	60 (30.8)	
≥ 6,640.00	5,269 (35.5)	64 (32.8)	
Private health insurance (%)	10,160 (68.2)	138 (70.5)	0.57
Hypertension (%)	5,268 (35.4)	132 (67.0)	< 0.0001
Controlled hypertension (%)	12,948 (86.9)	153 (77.7)	< 0.0001
Diabetes mellitus (%)	2,901 (19.5)	68 (34.5)	< 0.0001
Dyslipidemia (%)	8,647 (58)	128 (65)	0.049
Controlled dyslipidemia (%)	7,603 (51)	114 (57.9)	0.057
Chronic kidney disease (%)	943 (6.3)	33 (16.8)	< 0.0001
Atrial fibrillation or flutter (%)	48 (0.3)	0 (0)	0.42
Smoking (%)			
Never	8,495 (57.0)	99 (50.3)	0.16
Past	4,462 (29.9)	69 (35.0)	
Current	1,947 (13.1)	29 (14.7)	
Alcohol consumption (%)			
Never	1,587 (10.7)	27 (13.8)	< 0.0001
Past	2,973 (20.0)	61 (31.1)	
Current	10,320 (69.3)	108 (55.1)	
Body mass index (kg/m²)*	27.0 ± 4.8	27.1 ± 4.6	0.79
Physical activity at leisure (%)			
Inactive	9,287 (63.2)	135 (69.9)	0.15
Partially active	1,851 (12.6)	21 (10.9)	
Active	3,554 (24.2)	37 (19.2)	
CIMT* (mm)	0.92 ± 0.20	1.03 ± 0.24	< 0.0001
AAS	820 (5.5)	145 (73.6)	< 0.0001
Other antiplatelets	72 (0.5)	14 (7.1)	< 0.0001
All antiplatelets	847 (5.7)	60 (30.5)	< 0.0001
Antivitamin K	50 (0.3)	6 (3)	< 0.0001
Heparin	4 (0.0)	1 (0.5)	< 0.0001
Antihypertensives	4,383 (29.4)	134 (68)	< 0.0001
Number of antihypertensive medications			
Only 1	2,051 (13.8)	54 (27.4)	< 0.0001
2	1,650 (11.1)	52 (26.4)	
3 or more	682 (4.6)	28 (14.2)	
Diuretics	2,226 (15.0)	66 (33.5)	< 0.0001
Beta blockers	1,576 (10.6)	45 (22.8)	< 0.0001
ACEI	1,582 (10.6)	56 (28.4)	< 0.0001
ARB	1,192 (8.0)	44 (22.3)	< 0.0001
Calcium channel blockers	780 (5.2)	27 (13.7)	< 0.0001
Vasodilators	72 (0.5)	6 (3)	< 0.0001
Centrally acting antihypertensive	61 (0.4)	8 (4.1)	< 0.0001
Alpha blockers	36 (0.2)	0 (0)	0.49
Aliskiren	8 (0.1)	1 (0.5)	0.01
Cholesterol-lowering drugs	1,915 (12.9)	63 (32.0)	< 0.0001
Statins	1,764 (11.9)	59 (29.9)	< 0.0001
Controlled LDL levels	7,243 (49)	43 (42.1)	0.06
Adherence to use of any medication	37.1	46	0.02

CIMT = carotid intima-media thickness; AAS = acetylsalicylic acid, ACEI = angiotensin-converting enzyme inhibitors; ARB = angiotensin receptor blockers. *Data are presented as means (± standard deviation).

In the bivariate analysis, the participants with stroke reported making greater use of aspirin, other antiplatelet drugs and anticoagulants (antivitamin K) (Table 2), compared with non-stroke participants (Table 2). Although there was no difference in the frequency of stroke according to sex, it was noticed that the men made greater use of aspirin (respectively, 59.6% versus 40.4%; P = 0.02) and greater use of other antiplatelet drugs (respectively, 71.4% versus 28.6%; P = 0.04), and that there was a trend towards greater use of antivitamin K drugs (respectively, 83.3% versus 16.7%; P-value = 0.056), compared with the women. Having private health insurance was not associated with higher use of other antiplatelet drugs.

**Table 2.** Use of main antiplatelet and anticoagulant drugs in secondary prevention of stroke

	AAS		P-value	Other antiplatelets		P-value	Antivitamin K		P-value
	No	Yes		No	Yes		No	Yes	
	n = 145 (%)	n = 52 (%)		n = 183 (%)	n = 14 (%)		n = 191 (%)	n = 6 (%)	
Age (years)*	58 (8.7)	59.4 (9.5)	0.37	57.9 (8.6)	64.5 (9.9)		58.2 (8.9)	64.5 (6)	0.09
Sex (%)									
Male	58 (40)	31 (59.6)	0.02	79 (43.2)	10 (71.4)	0.04	84 (44.0)	5 (83.3)	0.056
Female	87 (60)	21 (40.4)		104 (56.8)	4 (28.6)		107 (56.0)	1 (16.7)	
Race (%)									
White	60 (42.3)	28 (54.9)	0.58	80 (44.4)	8 (61.5)	0.78	84 (44.7)	4 (80.0)	0.60
Mixed	47 (33.1)	12 (23.5)		56 (31.1)	3 (23.1)		58 (30.9)	1 (20.0)	
Black	28 (19.7)	9 (17.6)		35 (19.4)	2 (15.4)		37 (19.7)	0 (0.0)	
Asian	5 (3.5)	1 (2)		6 (3.3)	0 (0)		6 (3.2)	0 (0.0)	
Indigenous	2 (1.4)	1 (2)		1 (1.7)	0 (0)		3 (1.6)	0 (0.0)	
Education (%)									
Less than high school	34 (23.4)	14 (26.9)	0.87	44 (24)	4 (28.6)	0.23	47 (24.6)	1 (16.7)	0.41
High school and some college/university	51 (35.2)	18 (34.6)		67 (36.6)	2 (14.3)		68 (35.6)	1 (16.7)	
College/university or more	60 (41.4)	20 (38.5)		72 (39.3)	8 (57.1)		76 (39.8)	4 (66.7)	
Mean monthly family income (%)									
≤ USD 1245	54 (37.8)	17 (32.7)	0.75	68 (37.4)	3 (23.1)	0.49	71 (37.6)	0 (0.0)	0.11
USD 1246 to 3319	44 (30.8)	16 (30.8)		56 (30.8)	4 (30.8)		58 (30.7)	2 (33.3)	
≥ USD 3320	45 (31.5)	19 (36.5)		58 (31.9)	6 (46.2)		60 (31.7)	4 (66.7)	
Private health insurance (%)	103 (71)	35 (67.3)	0.62	125 (68.3)	13 (92.9)	0.053	133 (69.6)	5 (83.3)	0.47

AAS = acetylsalicylic acid; USD = United States dollars.

Table 3 shows the main classes of anti-hypertensive drugs used for stroke secondary prevention: diuretics, beta-blockers, angiotensin-converting enzyme inhibitors (ACEI), angiotensinogen receptor blockers (ARB) and calcium channel blockers (CCB). The most frequently used antihypertensive medication in the sample was diuretics, followed by ACEI, β-blockers, ARB, CCB, centrally acting hypertensives and vasodilators. No difference in the use of antihypertensive drugs was found between men and women, except for ACEI, for which there was greater use among men than among woman (57.1% versus 42.9%; P-value = 0.03).

**Table 3.** Main classes of antihypertensive drugs used for secondary prevention of stroke

	Diuretics		P	Beta-blockers		P	ACEI		P	ARB		P	Calcium channel blockers		P
	No	Yes		No	Yes		No	Yes		No	Yes		No	Yes	
	n = 131(%)	n = 66 (%)		n = 152 (%)	n = 45 (%)		n = 141 (%)	n = 56 (%)		n = 153 (%)	n = 44 (%)		n = 170 (%)	n = 27 (%)	
Age (years)*	57.2 (8.4)	60 (9.4)	0.009	58 (8.7)	60 (9.3)	0.22	58.3 (9.1)	58.6 (8.4)	0.81	57 (8.9)	63 (7.2)	< 0.0001	57.7 (9)	62.8 (6.9)	0.005
Sex (%)															
Female	72 (55.0)	36 (54.5)	0.96	86 (56.6)	22 (48.9)	0.36	84 (59.6)	24 (42.9)	0.03	86 (56.2)	22 (50.0)	0.47	95 (55.9)	13 (48.1)	0.45
Male	59 (45)	30 (45.5)		66 (43.4)	23 (51.1)		57 (40.4)	31 (57.1)		67 (43.8)	22 (50.0)		75 (44.1)	14 (51.9)	
Race (%)															
White	66 (52.0)	22 (33.3)	0.006	68 (45.6)	20 (45.5)	0.23	65 (47.1)	23 (41.8)	0.62	72 (48.3)	16 (36.4)	0.27	79 (47.3)	9 (34.6)	0.04
Mixed	31 (24.4)	28 (42.4)		49 (32.9)	10 (22.7)		38 (27.5)	21 (38.2)		44 (29.5)	15 (34.1)		51 (30.5)	8 (30.8)	
Black	25 (19.7)	12 (18.2)		26 (17.4)	11 (25.0)		29 (21.0)	8 (14.5)		25 (16.8)	12 (27.3)		30 (18.0)	7 (26.9)	
Asian	5 (3.9)	1 (1.5)		5 (3.4)	1 (2.3)		4 (2.9)	2 (3.6)		6 (4.0)	0 (0.0)		6 (3.6)	0 (0.0)	
Indigenous	0 (0.0)	3 (4.5)		1 (0.7)	2 (4.5)		2 (1.4)	1 (1.8)		2 (1.3)	1 (2.3)		1 (0.6)	2 (7.7)	
Education (%)															
< High school	27 (20.6)	21 (31.8)	0.21	39 (25.7)	9 (20.0)	0.32	30 (21.3)	18 (32.1)	0.19	37 (24.2)	11 (25.0)	0.43	37 (21.8)	11 (40.7)	0.10
High school/ some college/university	47 (35.9)	22 (33.3)		49 (32.2)	20 (44.4)		49 (34.8)	20 (35.7)		57 (37.3)	12 (27.3)		62 (36.5)	7 (25.9)	
College/university or more	57 (43.5)	23 (34.8)		64 (42.1)	16 (35.6)		62 (44.0)	18 (32.1)		59 (38.6)	21 (47.7)		71 (41.8)	9 (33.3)	
Mean monthly family income (%)															
≤ USD 1245	44 (34.1)	27 (40.9)	0.50	55 (36.7)	16 (35.6)	0.90	49 (35.3)	22 (39.3)	0.52	59 (39.1)	12 (27.3)	0.20	60 (35.5)	11 (42.3)	0.74
USD 1246 to 3319	43 (33.3)	17 (25.8)		47 (31.3)	13 (28.9)		41 (29.5)	19 (33.9)		47 (31.1)	13 (29.5)		52 (30.8)	8 (30.8)	
≥ USD 3320	42 (32.6)	22 (33.3)		48 (32.0)	16 (35.6)		49 (35.3)	15 (26.8)		45 (29.8)	19 (43.2)		57 (33.7)	7 (26.9)	
Private health insurance (%)	97 (74.0)	41 (62.1)	0.09	105 (69.1)	33 (73.3)	0.58	107 (77.9)	31 (55.4)	0.005	100 (65.4)	38 (86.4)	0.007	120 (70.6)	18 (66.7)	0.68

ACEI = angiotensin-converting enzyme inhibitors; ARB = angiotensin receptor blockers.

Lower frequency of diuretic use was observed among whites with stroke and higher frequency of diuretic use was observed among mixed-race participants with stroke, compared with others (P = 0.006). Furthermore, lower frequency of CCB use was noticed among whites with stroke and higher frequency of CCB use among black participants with stroke, compared with others without this condition (P = 0.04).

Regarding educational attainment, use of centrally acting antihypertensive medication was higher among stroke participants with less education than among participants with higher levels of education. Having private health insurance was associated with higher use of ARB among stroke participants, compared with others.

Use of statins was also greater among older participants (P < 0.0001) and among men in comparison with women (64.4% versus 35.6%; P < 0.0001). Stroke participants reported making greater use of statins, compared with non-stroke participants. The frequency of use of statins was higher among white participants with stroke, compared with others (P = 0.04) (Table 4).

**Table 4.** Characteristics of participants with stroke according to use of statins

	Use of statins		
	No	Yes	P-value
	n = 138 (%)	n = 59 (%)	
Age (years)*	56.7 (8.7)	62.3 (8.2)	< 0.0001
Sex (%)			
Male	51 (37.0)	38 (64.4)	< 0.0001
Female	87 (63.0)	21 (35.6)	
Race (%)			
White	54 (39.7)	34 (59.6)	0.04
Mixed race	45 (33.1)	14 (24.6)	
Black	31 (22.8)	6 (10.5)	
Asian	3 (2.2)	3 (5.3)	
Indigenous	3 (2.2)	0 (0.0)	
Years of education (%)			
< 9	35 (25.4)	13 (22.0)	0.44
9-11	51 (37.0)	18 (30.5)	
> 11	52 (37.7)	28 (47.5)	
Mean family monthly income (%)			
≤ USD 1245	55 (40.1)	16 (27.6)	0.11
USD 1246 to 3319	43 (31.4)	17 (29.3)	
≥ USD 3320	39 (28.5)	25 (43.1)	
Private health insurance (%)			
No	44 (31.9)	15 (25.4)	0.37
Yes	94 (68.1)	44 (74.6)	

Table 5 shows the use of medication according to the time that had elapsed since the stroke. Absence of use of medication increased with increasing length of time since the stroke, such that around 20% of the participants for whom 10 years or less had elapsed since their stroke were not using medication and around 30% of the participants who reported that 11 years or more had elapsed since their stroke were not using medication. Overall, the use of medication for secondary stroke prevention was very low in this sample and it declined with increasing length of time since the stroke. The percentage of the participants who reported using at least one antiplatelet or anticoagulant drug in association with antihypertensives and statins was around 15% to 20%.

**Table 5.** Medication use according to drug classes and length of time since occurrence of stroke, among participants with self-reported stroke

Drug classes and combinations	Time since stroke (years)				Total
	n (%)				
	< 5	5-10	11-15	> 15	
No medication use	14 (19.4)	8 (16.7)	11 (35.5)	12 (30.8)	45 (23.7)
Only aspirin	3 (4.2)	2 (4.2)	2 (6.5)	0 (0)	7 (3.7)
Only antihypertensives	17 (23.6)	22 (45.8)	10 (32.3)	14 (33.3)	63 (33.2)
Only statins	3 (4.2)	0 (0)	1 (3.2)	3 (25.8)	7 (3.7)
Aspirin and any antihypertensive	9 (12.5)	3 (6.3)	2 (6.5)	2 (7.7)	16 (2)
Aspirin and statins	2 (2.8)	0 (0)	0 (0)	0 (0)	2 (1.1)
Other antiplatelets and antihypertensives	2 (2.8)	0 (0)	0 (0)	0 (0)	2 (1.1)
Aspirin, other antiplatelets and statins	0 (0)	1 (2.1)	0 (0)	0 (0)	1 (1.1)
Antihypertensives and statins	7 (9.7)	5 (10.4)	1 (3.2)	1 (2.6)	14 (7.4)
Aspirin, antihypertensives and statins	12 (16.7)	3 (6.3)	2 (6.5)	3 (7.7)	19 (10.5)
Antivitamin K, antihypertensives and statins	1 (1.4)	1 (2.1)	1 (3.2)	0 (0)	3 (1.6)
Other antiplatelets, antihypertensives and statins	1 (1.4)	0 (0)	0 (0)	3 (7.7)	4 (2.1)
AAS, antiplatelets, antihypertensives and statins	1 (1.4)	3 (6.3)	1 (3.3)	1 (2.6)	6 (3.2)
Total	72	48	31	39	190

## DISCUSSION

Overall, the use of medication was very low among these participants in ELSA-Brasil, despite the existence of cheap medications such as aspirin or β-blockers, and a national policy that guarantees access to essential medications for all citizens.

Although the frequencies of stroke according to sex were similar (no statistical difference), the men reported making greater use of medication for secondary prevention of stroke than did women. Recent data on the prevalence of stroke from the National Health Survey (Pesquisa Nacional de Saúde, PNS) and from the Global Burden of Disease Brasil study have also shown similar results.^3^^,^^6^ If the prevalence is similar between the sexes, and considering that women use healthcare services more frequently than men do,^14^^,^^15^^,^^16^ it might have been expected that women would have higher use of medication than men.

The data from ELSA-Brasil showed that the prevalence rates regarding awareness about being hypertensive, use of medication and control of high blood pressure were higher among women than among men. The same was true for use of statins. One possible explanation is that participants who reported having had a stroke presented lower mean monthly family income than did non-stroke participants. Moreover, from analysis on the data according to sex, it could be seen that women who reported having had a stroke presented lower income than did men who reported having had a stroke. Discussion of adherence to treatment is also a complex matter since it is likely that some participants, especially women, had not had any treatment prescribed and thus were not taking any medication. Therefore, our results are similar to those from a previous Brazilian study that reported that there was only low use of medication for chronic diseases.^17^

Our data highlighted some important points. ELSA-Brasil participants are civil servants with higher levels of education, higher mean monthly family income and better access to medical services, compared with the general Brazilian population. However, even considering this better access to medical services, the frequency at which participants who had had a stroke reported using antiplatelet or anticoagulant drugs in association with antihypertensives and statins was never higher than 21%. Nonuse of medication was less than 20% among individuals who had had a stroke ten years or less before their baseline interview, but this rose to more than 30% among those whose stroke was more than 10 years earlier. Frequency of use of medication also declined according to the length of time since the stroke.

Brazil has implemented a national drug policy to guarantee access to essential drugs for all citizens.^18^ Drugs used for secondary prevention of stroke are in the list of essential drug products (RENAME).^19^ The Ministry of Health has also created the Brazilian Popular Pharmacy Program (FPB)^20^ with the aim of increasing access to essential and primary medicines for all citizens.^21^^,^^22^

Although these drugs should be available for all individuals, some studies have indicated that not all antiplatelet or antihypertensive medications are always available through public healthcare system.^23^^,^^24^ It is important to note that at the time of the baseline examinations in ELSA-Brasil (between August 2008 and December 2010) only two antihypertensive medications were included in the Popular Pharmacy Program: beta blockers and ACEI. In 2010, statins and ARB were also included, and aspirin was added in 2011. Although our data did not show any difference in the use of secondary prevention according to mean monthly family income, it would be interesting to investigate the extent to which secondary prevention was being used at the time of the second ELSA-Brasil examination (conducted between 2012 and 2014). At that time, all the drugs used for prevention were available through all programs.

The pattern of statin use that we observed is similar to that presented in the Reasons for Geographic and Racial Differences in Stroke Study (REGARDS). Data from that study showed that the use of medication was highest among white men followed by the levels among black men, white women and black women.^25^ One potential explanation for the more aggressive use of statins among white men relative to the other groups may lie in physicians’ treatment patterns and habits.^25^ In 2004, Mosca et al. found that although primary care physicians widely supported the National Cholesterol Education Program’s Third Adult Treatment Panel guidelines and coronary heart disease (CHD) risk stratification, less than half of these physicians implemented risk calculation tools in their clinical practice. This resulted in consistent underestimation of CHD risk, especially among women.^26^

Having private health insurance was associated with higher frequency of ARB prescription than of ACEI prescription and a borderline trend towards use of antiplatelet drugs other than aspirin, along with higher frequency of use of centrally acting antihypertensive drugs. These data suggest that participants with private health insurance are using less cost-effective and more expensive antihypertensive and antiplatelet medications, in comparison with participants without private health insurance. Although no information regarding the incidence of the main stroke subtypes (ischemic or hemorrhagic) was available from ELSA-Brasil, no participant who self-reported having had a stroke in this sample presented atrial fibrillation or flutter. This may explain the low use of anticoagulants in the sample.

Stroke is a preventable disease. There is now a growing body of evidence highlighting the importance of comprehensive risk factor management for improving survival among stroke participants, especially through detection, treatment and control of high blood pressure.^27^ Despite the good results from hypertension treatment that were achieved in one selected sample,^28^ a recent meta-analysis based on ten Brazilian cross-sectional studies^29^ estimated that in Brazil in the 2000s, only 24.1% of the cases of high blood pressure were kept under control. Although this percentage was comparable to that of other countries,^30^^,^^31^^,^^32^ it was still low.

Some authors have adopted the concept of epidemiological transition to describe trends among cardiovascular diseases. Briefly, there was firstly a shift from rheumatic heart disease to hypertensive disease and hemorrhagic stroke. In the next stage, there was a shift from hemorrhagic to ischemic stroke, and finally to CHD among middle-aged individuals. In the last stage, there was a switch from stroke to CHD mortality.^32^ Brazil is in the last stage of this epidemiological transition. Around ten years ago (2007), ischemic heart diseases surpassed stroke as the most prevalent cause of death in this country. However, the burden of stroke is still very high and is associated with premature death.

In this sample from ELSA-Brasil, which had better access to healthcare and higher monthly income than that of the general population of Brazil, the frequency of use of secondary prevention was very low. This finding reaffirms that stroke is still a neglected disease in Brazil,^34^ and that this scenario has not changed over the last 10 years.^33^^,^^34^

This study had several limitations. The most important of these was that information about stroke in the baseline examination was self-reported, based on reports of previous medical diagnoses of stroke. Therefore, there was no information either about the frequency of the main subtypes of stroke (ischemic and hemorrhagic) or any confirmation of the diagnosis of stroke through medical records or imaging.

Recently, a study calculated the specificity of self-reported diagnoses of stroke using hospital data as the gold standard. It reported values of around 99%, but with low sensitivity.^35^ In epidemiological studies, it is preferred to use questionnaires with greater specificity than sensitivity. Thus, in our study, self-reported occurrences of stroke were most likely true cases. However, milder cases that might have corresponded to minor stroke were likely to have remained undiagnosed as stroke. Thus, such cases would not be reported by ELSA-Brasil participants and, consequently, they are not included in this analysis. Therefore, it is still possible that there may have been some misclassification of cases of stroke in our study.

Interestingly, higher frequencies of risk factors and higher CIMT values were found among stroke participants than among other individuals without this condition, thus suggesting that the self-reported information indeed had value. Furthermore, detailed information about the participants’ use of medication was available, and this provided an insight into the situation of secondary prevention of stroke in this sample. However, we did not have access to the medical reasons for prescribing specific medications to specific patients. Moreover, the eligibility criteria for ELSA-Brasil excluded individuals with communication problems or with cognitive deficits, which probably excluded some stroke cases. Hence, cases of stroke in ELSA-Brasil were probably milder cases with less incapacity than what has been seen in other samples.

Discussion of adherence to treatment is also a complex matter since it is likely that some participants, especially women, had not been prescribed any treatment and thus were not taking any medication. Thus, our results are similar to those of a previous Brazilian study that reported low use of medications for chronic diseases.^17^ Considering that the ELSA-Brasil sample had a higher educational level, higher mean monthly family income and better access to healthcare services, the overall picture for the general population in Brazil is probably worse.

## CONCLUSIONS

In the ELSA-Brasil, the frequency of use of secondary prevention for stroke was low and decreased as the length of time since the stroke increased. Moreover, sex was the variable most associated with use of secondary prevention in this sample. Men reported greater use of medication for secondary prevention of stroke than women, thus suggesting that the rate of prescription of medications for secondary prevention of stroke was low among woman.
